# Municipal healthcare professionals’ interprofessional collaboration during older patients’ transitions in the municipal health and care services: a qualitative study

**DOI:** 10.1186/s12913-022-08226-5

**Published:** 2022-07-15

**Authors:** Marianne Eika, Sigrun Hvalvik

**Affiliations:** 1 Faculty of Health- and Social Sciences, Institute of Nursing and Health, University of South-Eastern Norway, Kjoelnes Ring 56, 3918 Porsgrunn, Norway; 2 USN Research Group of Older Peoples’ Health, University of South- Eastern Norway , Kjoelnes Ring 56, 3918 Porsgrunn, Norway

**Keywords:** Municipality, Interprofessional collaboration, Transition, Older patients, Prioritisations

## Abstract

**Background:**

Interprofessional collaboration is vital to assist patients towards a healthy transition in the municipal health and care services. However, no study has so far investigated municipal health care providers’ inter-professional collaboration during older patients’ transition in the municipal health and care services. The aim of this study is therefore to describe and explore what influence health care providers’ inter-professional collaboration within and across municipal facilities during older patients’ transitions in the municipal health and care services.

**Method:**

The study has a descriptive, interpretive design. Focus group interviews and individual interviews with municipal health care providers different professions were performed.

**Results:**

Municipal health care providers’ inter-professional collaboration during older patients transitions in the municipal health and care services was challenging. Two main themes were identified: The patient situation itself and Professional. Personal, and Practical circumstances. The results show that the municipal priority of patients staying at home as long as possible facilitated inter-professional collaboration across the short-term care facility and the home care services. Inter-professional collaboration across facilities with the long-term care facility was downgraded and health care providers in this facility had to cope as best they could.

**Conclusion:**

Prioritising and facilitating inter-professional collaboration between the short-term care facility and the home care services, contributed to health care providers experiencing doing a proper and safe patient assistance. Yet, this priority was at a cost: Health care providers in the long-term care facility, and in particular registered nurses felt squeezed and of less worth in the municipal health and care services. It was a strain on them to experiencing unplanned and often rushed patient transition into long-term care facility. To focus on municipal inter-professional and inter-facility collaboration during patients in transition to long-term care placement is vital to maintain the patients, and the health care providers working in these facilities.

**Supplementary Information:**

The online version contains supplementary material available at 10.1186/s12913-022-08226-5.

## Background

Interprofessional collaboration (IPC) is decisive to secure seamless and comprehensive patient care pathways [1–4]. Older persons may experience concurrent transitions in later life, and municipal health care providers in different contexts and settings may play significant roles to ensure a healthy and safe transition for the patients and their next of kin [5–7]. Healthy transitions include subjective well-being, well-being of relationships, and role mastery. The focus of this study is on what influence municipal health care providers’ IPC during older patients’ transitions in the municipal health and care services.

Demographic challenges, with an increased number of older people [2, 8], and limited municipal resources [9] require resources used more effectively [10]. After the implementation of the Coordination reform in 2012 in Norway, the municipal health and care services have greater responsibilities to ensure that vital health and care services are delivered to the population [9, 11]. Older patients are discharged from hospital quicker and sicker than before [12] and require coordinated health and care services across health care levels [12–14]. Municipal nursing home-and home-care services have become more specialised [13, 15, 16], and studies observe a shift towards more medical focus at the cost of holistic care [13, 17, 18].

Providing comprehensive healthcare to older patients with complex needs is difficult and multifaceted [4, 19]. Older patients are particularly exposed to risks during transitions due to multimorbidity, complex and diffuse symptoms and circumstances [2, 15, 16]. The patients may have to move between different municipal health care locations and health care professionals [20, 21], as their health declines, and their next of kin may need respite or are unable to support their older family member at home.

National health policy advocates patients live at home longer [2, 9, 11]. Efforts are made to avoid admitting older persons to hospital by addressing their treatment and care needs in the municipal health and care services [2, 9]. Treatment of patients’ diseases within the municipality is critical, as well as having enough competent licensed health care providers at work ([21], p.14). Furthermore, clinical practices show that interprofessional and inter-sector teams contribute to postponing and preventing nursing home admissions as well as hospital admissions [2]. At some point in older patients’ health and care trajectory, however, they may need institutional care. National authorities emphasize that municipal health policy should support smoother relocations and transitions between home and nursing home to maintain and increase continuity, safety and predictability for older patients, and better respite, support, and further involvement for next of kin. Moreover, the authorities suggest longer exchange fields [9], where health care providers at the facilities overlap and “run parallel” for some time (p. 158). Studies exploring planned relocations to nursing homes are needed ([21], p 11).

In this study, we understand IPC as “integrative cooperation of different health professionals, blending complementary competence and skills, to the benefit of the patient, making possible the best use of resources in a primary care setting” ([22], p. 305), at personal as well as system level.

Transition is a complex concept, and the definition of transition varies according to the disciplinary focus [23]. One frequently used definition is that it is a “passage from one life phase, condition or status to another, a multiple concept embracing the elements of process, time span and perception” ([24], p.25-26]). There are several types of transitions including situational, developmental and health and illness [25, 26]. In this study we do not differentiate between types of transition as municipal health care providers must relate to them all. Moreover, as transitions related to both changes in patients’ health status and situations may involve care transitions, this study focuses mainly on IPC during older patients’ care transitions. Care transitions include a wide view on interprofessional and interorganisational interaction, linked to patient movement across care settings [27]. According to Aase and colleagues, quality in care transitions “encompasses patient-centered, communicative, collaborative, cultural, competency-based, accountability-based, and spatial dimensions to ensure interactions among patient and next of kin, health care professionals and organisations as patients move across settings ([27], p. 11). In this study the concepts “transition” and “care transition” are used interchangeably because care transitions often involve transition experiences for patients.

We understand health care organisations as complex [28–31]. Older patients’ transitions and care transitions occur in complex circumstances and contexts where different health care providers, within and between municipal health care facilities are responsible for different aspects of patient care and treatment. According to Waring and Aase [31], healthcare in complex adaptive systems is characterised by variability, non-linearity, and complex interactions, “which together place extraordinary demands on the stakeholders involved in the quality of care transitions” (p. 286).

Municipal health care providers may facilitate or impede the transition process during care transitions. We have been unable to find studies undertaken in a Norwegian context exploring what influence municipal health care providers’ IPC specifically focusing on older patients’ transition in the municipal health and care services. However, several studies have explored what influence IPC within and across health care sectors [3, 21, 32–37]. The studies have multiple perspectives and focus on interactions between health care providers in diverse settings. Some studies have found barriers as well as facilitators to IPC at organizational and individual levels. For instance, IPC across all contexts was hampered by geography, organizational and individual factors, including financial conditions, differences in professional power, knowledge bases, and professional culture [3, 21, 38]. In a review study exploring IPC in municipal care as perceived by involved actors [39], main facilitators to IPC were that the different actors shared interest in collaboration and perceived opportunity to improve quality of care. Main barriers were “the challenges of definition and awareness of each other’s roles and competence, shared information, confidentiality and responsibility, team building and inter-professional training” (p. 716). The CrossCare Old study in Norway [40], explored quality in cross-sectoral care transitions for older patients moving between various services, some of which explored quality between municipal services. According to a meta-analysis of studies in this project [41], the main theme “Vulnerable coordination in the health and care services for the elderly” was linked to the “interplay between personal needs and preferences, the social context and the service context” (p.10). The authors conclude that the challenges relate to a pressured health care system, with large workloads, lack of competence, and capacity. Moreover, the informants called for structure and management support, as well as health care policy that supports continuity of care.

## Methods

### Aim

This study aimed at exploring and describing what influenced municipal health care providers’ IPC during older patients’ transitions in the municipality.

### Design

The study has a qualitative approach with a descriptive and explorative design because little is known from former research studies about what influences IPC during older patients’ transition in the municipal health and care services.

Two data sources were used: focus group interviews and individual interviews.

### Setting and context

The current study focused on the three most common care transitions in the municipal health and care services. A: Transition from short-term care facility (STCF) to home care services (HCS), B: Transition from HCS to STCF; and C: from STCF to long-term care facility (LTCF).

The municipal policy is to prioritize patients to stay in their homes as long as possible. Health care providers in the STCF, the HCS, and the physiotherapy services, had scheduled regular IPC meetings between the STCF and the HCS. The general practitioner (GP) employed part-time at the STCF did not participate at these meetings because the GP was not at work on the weekday these meetings were arranged. There were no organized IPC meeting points between the STCF and the LTCF during patients’ transition to LTCF.

The admission team meeting assesses applicants’ needs and allocates services to the HCS or nursing home services. At the time of this study, the admission team members were facility/zone leaders of the nursing- and physiotherapy services (except the LTCF), one GP employed part-time at the nursing home, and a secretary.

Each facility had its own nursing staff, apart from some unlicensed assistants who worked across municipal facilities. The nursing staff comprise registered nurses, auxiliaries, and unlicensed assistants. The latter have no formal health care education. At the time of this study, assistants worked at the STCF at weekends only. At the HCS and LTCF assistants worked daily.

The physiotherapists, the occupational therapist and the physicians worked across institutions. At the nursing home, the GPs employed at the different facilities, combined working part-time with full-time employment at the municipal medical centre. The majority of GPs are self-employed, with private contracts with municipalities, to serve these services part-time [3, 38]. The occupational therapist was newly employed at the time of the interview and provided few data to the study.

### Recruitment and participants

This study was a follow- up study of a doctoral study [28], conducted in a medium-sized rural municipality located in South-Eastern Norway.

Municipal health care providers at different professional and functional levels suggested possible participants for the focus group interviews in collaboration with first author. We decided to have three heterogenous focus groups to attempt diversity and to facilitate reflection among the participants regarding IPC [42]. The sample was purposive with participants sharing some similar characteristics, and who had something to say about the topic [43]. Six to nine health care providers participated in each focus group. The participants were registered nurses, auxiliaries, assistants, physiotherapists, one occupational therapist, and general practitioners. The participants in one focus group were facility leaders of the nursing-, physiotherapy - and occupational therapy services, in addition to physicians. The participants in the two other groups were front-line staff: a mix of nursing staff (licensed and unlicensed) and physiotherapists across the in-and out services (Table 1).

**Table 1:** Characteristics of the focus groups

**Focus group 1,** **9 participants.** **Leader group (facility leaders of the physiotherapy-, occupational-, and nursing services), general practitioners (GPs)** 4 registered nurses (2 in nursing home, 2 in home care services), 1 physiotherapist, 3 GPs (2 part-time employed in nursing home, 1 employed at the medical centre only), 1 occupational therapist (newly employed, left the meeting early)	**Focus group 2,** **8 participants.** **Front-line staff** 3 registered nurses, 1 physiotherapist, 2 auxiliaries, 2 assistants (5 in nursing home, 3 in home care services)	**Focus group 3,** **6 participants.** **Front-line staff** 1 physiotherapist, 1 registered nurse, 2 auxiliaries, 2 assistants (3 in nursing home, 3 across nursing home and home care services)

Although heterogeneity in the focus groups may contribute to bias the findings [43, 44], mixing health care providers may give valuable insights into group dynamics ([45], p. 103). It may also encourage getting to know each other’s roles and concerns across professions and municipal facilities and may thereby heighten the awareness of and advantage of IPC during older patients’ transition.

Mixing different levels of health care providers may contribute to uneven participation because in the professional hierarchy, some may display more authority and power [42] than others.

### Data collection

The semi-structured focus group interviews were in June 2018, and the individual interviews autumn 2018.

### Focus group interviews

Semi-structured interviews were conducted (Additional file 1). The interview guide comprised comparatively open-ended questions, which allowed the participants to share and discuss their various viewpoints and experiences. The interviews were led by first author (ME). Second author (SH) functioned as a second moderator in the two last interviews and secured that all topics were covered. After the first interview with the leaders at different municipal health care facilities, ME listened to the recording, transcribed the text, and shared some experiences and content with second author before the two last focus group interviews.

The interviews were in a separate room at the municipal nursing home at a convenient time for the participants.

### Individual interviews

After the focus group interviews were finished, first author had three individual interviews with one registered nurse, one General Practitioner, and one auxiliary. Based on the focus group interviews, these health care providers appeared central in further exploring what influenced IPC during older patients’ transition. These interviews lasted for ca. 1 h each and were performed at the health care providers’ workplaces at a convenient time for them.

### Data analysis  

The ontological position taken is constructivist with an analytic middle ground between reality and representation [46]. The analysis was inductive thematic [47] with focus on semantic as well as latent themes.

#### Focus group interviews

The interviews were audio-recorded and transcribed verbatim shortly after each interview by first author (ME). The texts were read several times to gain an overall impression [47]. Meaningful units were identified, coded, and categorized [47]. A search for similarities and differences between the categories was conducted, and overlapping categories were grouped together. The categories were formulated into sub-themes and finally into two themes [47]. Collaboration between the two researchers ensured nuances in the understanding and interpretation, and to reaching a consensus in the analysis process.

### Individual interviews

The individual interviews were undertaken after the focus group interviews. The interviews provided new data as well as additional data, which exemplified and complemented the main findings in the focus groups. An example of new data which influenced IPC was that specialized health care providers in the STCF facilitated IPC during older patients transition from the STCF to the HCS. Meaningful units describing what influenced IPC were extracted and categorized either as “facilitators” or “barriers” An example of a unit categorized under “facilitators” was health care providers expressed an attitude of keeping the patient safe during transition home. An example of a unit categorized under “barriers” was that nursing staff geographically distant from the medical centre, had difficulty getting in touch with the patient’s General Practitioner during transition. Subsequently, two main themes were developed (Additional file 2). By complementing the focus group interview data with the individual interview data, we were able to obtain broader and more nuanced insights into what influenced IPC during older patients transition in the municipality.

### Ethical considerations

The study followed the ethical principles outlined in the Declaration of Helsinki. The Data protection services at the Norwegian centre for research data (NSD) was notified about the project (59066). The study was considered outside the Norwegian Act of Medical and Health Research. Thus, it did not need an approval from the Regional Committees for Medical and Health Research Ethics. The health care authorities in the municipality gave formal access to the field. Written informed consent was obtained from all the health care providers who participated in the interviews, and all agreed to the interviews being recorded. Before the focus group interviews started, the participants were informed about the study orally, and they had the opportunity to ask questions before signing to confirm their informed consent. In addition, to underscoring each participants’ anonymity and their confidentiality, the participants were informed about their right to withdraw at any time without stating a reason.

## Findings

This study aimed at exploring and describing what influenced municipal health care providers’ IPC during older patients’ transitions in the municipality. Two distinct and closely related main themes were identified, each illuminating intertwining features of the complexity that challenged IPC during older patients’ transition in the municipality at inter- and intra-facility health and care levels. The themes: Patient situations that influenced IPC during older patients’ transitions in the municipality, and Professional, Personal, and Practical circumstances that influenced IPC during older patients’ transitions in the municipality.

### Patient situations that influenced IPC during older patients’ transitions in the municipality

The findings imply that patient situations influencing IPC had common features at intra- and inter-facility levels, related to prioritizations, expectations, relationships, and dilemmas.

The municipal priority of patients living at home longer, patient- and family situation and expectations, and professional knowledge, attitudes and standards influenced IPC.

To successfully manage the priority of patients with the potential of moving and staying at home, health care providers at the STCF and the HCS shared information and collaborated face-to face at formal and informal levels. I.e., as a rule, health care providers in the STCF informed the HCS about when a patient moved from the STCF back home (FG1, FG2). Moreover, health care providers in the HCS participated at decisive points in the patient’s trajectory during his/her stay at the STCF. I.e., the physiotherapist and occupational therapist collaborated with the nursing staff and the patient to investigate the need for installing helping aids in the patient’s home (FG1, FG2). Furthermore, licensed nursing staff at the HCS sometimes participated in direct care situations at the STCF, to better assist and support the patient at home. Often patients moved between municipal facilities, and health care providers in the HCS may have had previous experience with the patient now staying in the STCF. This encouraged the sharing of knowledge and experiences both ways when they met across these two facilities. This way, health care providers complemented each other’s knowledge and competence to ensure a healthy transition for the patients.

Individual auxiliaries and registered nurses specialised in different patient conditions. It was expected that they should share knowledge and skills with each other at formalised meetings and when needed. For instance, when a patient arrived in the STCF with a rehabilitation potential, the “specialist” rehabilitation nurse was primary nurse for that patient, if possible, and collaborated closely with the nursing staff and the physiotherapists.

When in doubt about whether the patient was capable of moving home and staying at home, health care providers within and across the facilities prioritised doing a proper job. I.e., licensed nursing staff, physiotherapists and physician collaborated closely with each other across the STCF and the HCS at three separate time intervals to assist the same patient to live at home (FG1, FG2). Moreover, prioritising patients’ psychosocial needs before moving home appeared a professional standard and a collective enterprise. Assisting patients could be time-consuming, and the findings suggest a culture of staff flexibility and willingness to help out so that the health care provider had sufficient time to support the patient properly:

*“I remember we had a patient who did not want to go outside of the facility, because she was afraid of meeting acquaintances in the neighbourhood. Therefore, we travelled to a nearby village to shops and such. It functioned well, and eventually she moved back home to see if she could manage. This worked out fine, and the transition was good at the end. It was a satisfying experience for us also, to see it was successful – it’s that safety issue, you know*” (FG2).

Different functions and interests at the facilities seemed to challenge IPC and create dilemmas. I.e., nursing staff in the HCS and the nursing home had different views about when a transition home was acceptable. Nursing staff at the nursing home underscored the importance of respecting the patients’ autonomy and that health care providers in the HCS be willing to live with uncertainty and unpredictability regarding patients who wanted to live at home:

“*Sometimes, the dangers are too much the focus* instead *of the possibility to try it out. Some patients prefer living at home half a year instead of staying here for three years”* (FG1).

In the HCS, the nursing staff were concerned about maintaining justifiable health care services when faced with unrealistic expectations from the patients, their next of kin and colleagues at the nursing home:

“*…that they believe they can move home and then there is 24- hour service, it is not like that when you live at home. It demands a little from, yes, next of kin and the patients themselves – that is how it is”* (FG1).

Some families expected and demanded considerable assistance from especially the nursing staff in the HCS regarding physically demanding patients. This stimulated relationships and alliances between the registered nurses and the patient’s General Practitioner (GP). The GP supported the nurses and together they argued against the patient moving home. Moreover, at the STCF, the findings indicate that shared interests and professional standards to protect the patients’ rights, stimulated IPC between the nursing staff and the GP. I.e., when next of kin overruled the patients and decided on behalf of them, the licensed nursing staff and the GP collaborated closely and developed coherent arguments to protect the patients’ rights:

*“The patient is our highest priority, and our focus has to be there. We cannot let the next of kin control too much as long as the patient is consent competent”* (individual interview).

Rushed care transitions from the HCS to the STCF due to sudden deterioration of patients’ health or next of kin’s health seemed to create some friction in the IPC between health care providers in the HCS and the STCF, because the “*information about the patient may be messier”* (FG2). During the focus group interview, the interaction between the nursing staff became tense regarding inadequate or lack of written information from the HCS. However, the participants quickly prevented further disagreement by the home care nurse’s agreeable solution: “…*just call us*” (FG2). in addition to reading documentation, e-messages, and consulting the patient and their next of kin, nursing staff in the STCF relied on getting up-dated information by phone from colleagues in the HCS.

Care transitions from the STCF to the long-term care facility (LTCF) could also happen quickly due to patient situation as well as circumstances. The nursing staff had little influence on this and were frustrated on behalf of the patient and next of kin:

“*It is a challenge to support the patient and the next of kin during that phase because, perhaps the more efficient the services are, maybe it is at the expense of next of kin and patients* (FG1).

Health care providers in the LTCF regarded moving into LTCF a stressful change and “*a very serious process for the patient concerned”* (FG1). However, they did not participate in the formalised IPC meetings across facilities. It disturbed the nursing staff that patients with a potential for moving back home was prioritised at the expense of patients moving into LTCF:

*“If you say yes to a patient, then you say maybe no to another patient… so one has to attempt a holistic approach”* (FG1).

Yet, sometimes when patients in spite of comprehensive IPC within and across the STCF and HCS were unable to live at home, this contributed to a planned transition to the LTCF. During the process of trying to live and manage at home, the patient realised and accepted that moving to LTCF was a better option:

*“With the support from us, she tried living at home again just to experience it. I believe the patient hoped to be able to stay in her home on a permanent basis. I think it was a difficult process for the patient to accept that she could not live at home any longer. Yet, she had had the opportunity to experience living at home. She appeared incapable to grasp this, but when she eventually moved into LTCF, I believe it was an ok process. A process many health care providers engaged in, both in the out-and in-services* (FG3).

### Professional, Personal, and Practical circumstances that influenced IPC during older patients’ transitions in the municipality

The professional, personal, and practical circumstances that influenced IPC during older patients’ transition were related to prioritisations, competence, roles, compensation, expectations, and local contexts. Hospital discharges, municipal policy, facilities and professionals with different functions and roles influenced IPC. Limited municipal resources paired with the priority of patients living at home longer appeared to impact less IPC between health care providers in the LTCF and the STCF and HCS.

The municipality has to receive patients from hospital at short notice. Then patients often moved from the STCF to the LTCF in a hurry, to provide a vacant bed for the new patient at the STCF. This seemed to have a negative impact on IPC. Nursing staff at both facilities were squeezed:

*“It is a challenge for us to assist and support everybody in this situation and be able to pass on information to the LTCF. I believe the reason is that we take this for granted because it is so common for us. Regarding the patient, this is once in a lifetime, so I think, perhaps it would have been better if we spent some more time on it. Yet, what is the most important here? Should we prioritize to spend the time we have on this? And then others must line up in another que to wait”* (FG1).

Nursing staff in the LTCF seemed frustrated because they had little influence on when the patients arrived from the STCF: “*we have to be allowed to decide that the patient arrive on Wednesday instead of Tuesday” (*FG1), and suggested:

“*We can be better at talking together, or that health care providers in the LTCF are present at the STCF and collaborate with the patient and colleagues to make the transition to LTCF easier for the patient. There is only one floor separating us”* (FG3).

They made efforts to make the patient and next of kin feel welcome despite the unfavourable circumstances:

**“…***at least we can show them that we think about this person and are well prepared. That the room is ready and that there is a name on the door, and that this particular person experiences that he/she is expected* (FG3).

The admission team meeting members appeared to collaborate closely with each other. The overview they had of the patients paired with the members’ different professional roles and work contexts, contributed to “heated discussions, negotiations and dialogues” (FG1). These interactions were considered a thorough base for making the right prioritizations when allocating services to patients. They seemed familiar with, and expected the potential benefits for the patients inherent at the different health care settings:

*I believe the admission team meeting is a good arena to support patients in transition - at least regarding new patients or patients who have lived at home and been to the nursing home - and see changes, and if they function better when they are going back home. At least, this is my experience, that they get another type of care (i*.e. improved nutritional-, functional- and social status) *during a period in the nursing home that help them function better at home”* (FG1)*.*

Decisions made at the meetings influenced IPC within and across facilities. As leaders of their respective facilities, they worked together with their front-line staff on weekdays. The findings suggest that front-line staff considered the information from the facility leaders assisted them, i.e. to “be able to take good care of the patient at home” (FG2). Equally, the admission team members acted on requests from their staff:

*«…. I think about being short of time…. I have occasionally participated in admission team meetings. The focus there is on healthy transition process. The members give room for extending the stay at the STCF, if we consider it necessary. You know, to support a safe and healthy transition for the patient. So at least, I think they try in many situations, to support the patients and their next of kin (*FG2).

Different facilities with different functions, professional resources and contexts influenced IPC. The municipal health and care services depend on using unlicensed assistants to have enough health care providers at work at any time. The findings demonstrate that it varied between the facilities how this influenced IPC.

At the LTCF, the licensed nursing staff worked side-by-side with their assistants during dayshifts weekdays (FG3). They then had opportunity to supervise and delegate tasks to the assistants and the assistants had the opportunity to ask questions. At weekends and nights, however, there were fewer licensed nursing staff at each shift, which limited close interaction between the unlicensed and licensed staff:

*“Especially when a new patient arrives, then I get nearly always information from the registered nurse and I get the rest myself, or ask the patient himself, what he can do. I experience I get all the information I need. Yet, when it is night or weekend and such – I can wait a little, but I ask the patient and it helps a lot”* (FG2).

At the HCS, nursing staff mostly operate on their own in the patients’ homes, and licensed nurses have restricted opportunity to work side-by-side with assistants. The findings suggest that geographical distances paired with professional and municipal policy, challenged IPC between licenced and unlicensed nursing staff. I.e., regardless of geographical distance, the licensed nurses did the tasks that required professional competence. This could mean too much time spent on driving between patients (FG2) instead of spending time with patients. Moreover, some assistants did not have password and access to the computer program (FG2). In these cases, the licensed nursing staff compensated, and wrote in their shift reports what the assistants reported to them. These circumstances created frustration among licensed nursing staff, and some tension in the collaboration with assistants. Moreover, it contributed to random and insufficient follow-up of patients in transition (FG2).

Due to limited physiotherapy services, two auxiliaries in the HCS had extended roles with a great degree of autonomy to i.e., compensate for the lack of physiotherapy services during patients’ transition home. They had regular supervisions from the physiotherapist regarding physical training and combined these skills with their competence as auxiliaries. The GP at the STCF, however, considered nursing staff incompetent regarding rehabilitation of stroke patients, and applied for rehabilitation stays outside the municipality.

The GPs’ involvement, expectations and roles concerning IPC varied. Some highlighted their focus was the patients’ medical condition and appeared to leave the rest to the other professional groups. Others seemed more involved with the patients’ transition and their next of kin. I.e. when next of kin insisted on supporting their older, multimorbid and cognitively impaired family member stay at home, the GP had a central role in supporting the next of kin and collaborated closely with health care providers in the HCS.

The findings hint that some GPs experienced being apart from IPC in the municipal health care services:

*“ …… at the medical centre, we get in a way a task or at least some inquiries from health care providers at the nursing home or the HCS. So, we in a way participate in those processes, either with medical information or multi dosage medications which need to be done something about. And if there is information the health care providers need, like patients’ journals”* (FG1).

At the medical centre, the GPs depended on and expected their colleagues in the HCS to do a proper mapping of the patient and next of kin situation: “T*hat we are told what the reality is concerning this patient…*.” (FG1). The findings imply some GPs regarded the communication and information sometimes lacking:

“*If the HCS is good at notifying us about someone needing more treatment and care, that we are being informed about this via nursing and care reports, or if they are ill and incapable of managing at home, that we are informed, or if we have to do something there and then, and then act from the information we have*” (FG1).

The findings indicate that the nursing staff in the HCS had difficulty getting in contact with the patient’s GP when in need, especially the GPs with most patients on their General Practitioner lists (FG2). It appeared especially challenging for the health care providers in the zone geographically further away from the medical centre to have a dialogue with the patient’s GP: “*We use a lot of time (*on the phone) *to get through to the GP*” (FG2). In the zone closer to the medical centre, it seemed the health care providers had easier access, and regular meeting points (individual interviews, FG2).

Health care providers appeared to recognise their own limitations when assisting patients in care transitions and regarded IPC essential:

*“….. the inter-professional part, that many professionals participate so that we get the views from several …it is very positive - to have good dialogues”* (FG2).

The formalized meeting points across the STCF and the HCS appeared to encourage licensed nursing staff and physiotherapists developing relationships and understanding of each other’s complementary roles. Similarly, keeping the patient safe during transition seemed paramount for most health care providers. They experienced that standing together made them feel they had done their best, and that their assistance provided safety for the patient, and peace of mind for themselves. Standing together implied a culture of flexibility, mutual dependency, and cognitive diversity where health care providers present shared their insights, worries, experiences, and knowledge about the patient with each other. Especially the physiotherapists expressed the need for close collaboration with the nursing staff at both facilities (FG1, FG2) to complement them in their assistance of patients with the potential of moving back home and staying at home.

The findings suggest, however, that IPC depended on group dynamics:

*“Regarding a good group, “chemistry” is important. I think, you can have loads of procedures and if it is somehow bad “chemistry” in the group, then things go slowly - there are huge differences how one gets it done”* (FG2).

In addition to face-to face IPC, health care providers shared information with each other via the computer program, telephone, and whiteboard. The findings indicate, however, that the written documentation in the computer program was not always read. Some facilities printed the information on paper, to ensure that everybody at the shift was updated on patient information (FG3).

Using the telephone to get updated information from colleagues appeared significant during older patients’ care transitions:

*“….. finally, it is the contact by phone to ensure that we are aware of the correct date - that is ok. Still, we do most of the communication and have a dialogue through x (computer program). The admission team meeting has prepared the transition beforehand, so that gives us insights into their assessment” (*FG2).

In the HCS, nursing staff and physiotherapists used whiteboards to inform and be updated on information about each patient and also each health care provider’s roles and functions at the shift. In addition to maintaining a consistent approach to the patient in transition, it seemed to encourage blending complementary competence and skills:

*“…… we have good descriptions of how to assist the patient that depend on what the patient’s aim is. And we have a dialogue with the patient, and work towards consistency in our approaches, and use the whiteboard in the rooms before we prioritize what is important for the patient to master, and the physiotherapists write exercises for the patients on the whiteboard and what we can do to assist in that respect. It is a very good tool to help us being updated. So, I agree with x that when roles are clear and everybody knows what to do, then it flows smoothly”* (auxiliary, FG2).

Across all facilities, the findings imply that the taken for granted challenged IPC. I.e., regarding patients in transition from the STCF to the LTCF, sharing information about the patient seemed at chance. These facilities were in the same building, and most health care providers were familiar with each other across the facilities. Just as this gave health care providers the opportunity to share information about the patient, it also seemed to contribute to the opposite; that health care providers ignored the sharing of what appeared to be obvious (FG1).

At the LTCF, little or lack of IPC with colleagues at the STCF and the HCS, seemed to contribute to some registered nurses feeling less worth. Their only collaborative partner was the part-time GP. Yet, at the focus group interviews, the GP appeared mainly focused on collaborating with GP colleagues and did not mention or talk about collaboration with the nursing staff (FG1). Likewise, the nursing staff did not talk about collaboration with the GP (FG3).

The findings indicate that being apart from IPC with the other facilities contributed to close intra-facility collaboration between the nursing staff all levels. Attitudes among them seemed to be to stick together and take as good care of the patients as possible:

*“When a new patient is expected at our facility, we in the group often talk together and plan the arrival. If some colleagues are good at something, then they can do that – we plan who will do what. I have noticed that things go smoothly then, and I (registered nurse) can focus on one thing instead of knowing that I have twenty other tasks to do. I have good help from the assistants then (*FG3).

The participants expressed that they thrived in the LTCF, which they believed contributed to patients feeling welcome: “*That you see, in a way, that the staff collaborate well together.”* (FG3).

At the STCF, the findings indicate that the facility aimed at a culture of generosity, where health care providers took advantage of each other’s strengths, and accepted “that some succeed while others do not” (individual interview) when assisting patients with multifaceted needs in transition. The findings imply clear roles and expectations between the staff. Moreover, the facility nurse regarded a good relationship with the GP employed at the STCF crucial to the quality of their work. The registered nurses knew what the GP expected them to map regarding patients in transition, which assisted the GP in efficient medical treatment and follow-up of the patients (Individual interviews). Similarly, the GP believed that the nursing staff and the physiotherapists were the main contributors during patients’ transitions. The GP at the STCF, however, expressed a need for closer collaboration with colleagues at the medical centre about plans they had made together with the patients, next of kin, and the HCS concerning the patients currently staying in the STCF.

## Discussion

The aim of this study was to describe and explore what influence municipal health care providers’ inter-professional collaborative (IPC) during older patients’ transitions in the municipality. The findings disclose two closely related main themes, each illuminating intertwining features of the complexity that challenged IPC during older patients’ transition at inter- and intra-facility health and care levels. The themes: Patient situations that influenced IPC during older patients’ transition and Professional, Personal, and Practical circumstances that influenced IPC during older patients’ transition.

Rushed hospital discharges challenged IPC between the STCF and the LTCF and contributed to unplanned transitions to the LTCF**.** If municipalities are unable to receive patients from hospital in need of municipal health and care services, the municipal authorities must pay a daily fee [3]. It demands courage from the municipal facility leaders to postpone discharges from hospital in an already pressurised municipal economy. As the finding suggest, patients eligible for a long-term care placement paid the price and had to move in a hurry to LTCF. Although health care providers at both facilities experienced this a dilemma, the rushed relocations went ahead. This seemed to cause some tension between health care providers at the STCF and LTCF and created a distance between them, which suggests a development towards less collaboration. Andersen et al. [48], also found that to save time influenced them to consequent risks of “disregarding important issues when treating and caring for elderly patients” (p. 2059). When professional standards of health and care services collide with what health care providers must adjust to, norms may shift. They may adapt to lower professional and ethical standards, especially regarding older patients in transition to long-term care placement. Not to prioritise older frail persons is contrary current political guidelines [2, 9]. Studies suggest that unplanned transitions into LTCF may result in severe consequences for the patients, such as anxiety, increased confusion, depression [49, 50], increased tendency of falls during the first 3months of residency [51], and it may stress next of kin [5, 52, 53].

That the members of the admission team meeting interacted regularly with their front-line staff, appeared to promote close IPC. The proximity between the admission team members and their front-line staff encouraged sharing of up-dated knowledge and efficient handling of matters, which contributed to maintaining continuity and consistency in their approaches to patients in transition between home and the STCF. This finding demonstrates the significance of proximity between those who access the needs for services and those who provide the services [54]. Studies show that distance between those who access the needs for services and those who provide the services inhibit IPC between levels of care [55, 56].

Limited municipal resources [57] challenged as well as stimulated IPC. Nursing staff at both the STCF and the LTCF expressed a gap between patients’ needs and what they could deliver when patients in transition to LTCF were not prioritised. Vike et al. [58], underscore that the dilemma of limited resources is decentralised to individual health care provider to handle. That the health care providers used discretion to maintain some inter-facility collaboration during older patients’ transition from STCF to LTCF appeared possible, due to the facilities being in the same building and health care providers’ relationships with each other. Yet, the current study suggests that the municipal policy contributed to limited possibility of using discretion, which worried the registered nurses. Equally, Ervik & Linden [59], found that health care providers in similar situations were concerned about the consequences for the patients as well as for the health care providers. According to the authors “monitoring and central control of service provisions is in opposition to the individual discretion of care work” (p. 2) with implications for service quality.

Transition is not a linear process [23] and in line with complexity science, the health care providers responded to unanticipated events [60], and patients’ fluctuating situations and needs. IPC across the admission team members, STCF, and HCS show that the health care providers lived with uncertainty and unpredictability regarding patients in transition home when they joined forces and assisted the same patient trying at three separate times to move back home. This suggests that health care providers in the HCS and the STCF were familiar with each other’s roles and functions. Especially the health care providers at the regular meetings expressed social ties, relationships, and trust. This experience is a precondition for knowledge flow ([61], p. 247), and may contribute to minimise the importance of professional, cultural, and organisational differences. Yet, prioritising some patients in this way, may indicate other mechanisms at play, such as the power of patients and next of kin to get their will through in this transparent rural municipality [5], and a desire among health care providers to do a proper job. That strong-willed patients who wished to move back home encouraged IPC, may have contributed to overshadowing vulnerable patients unable to speak for themselves [56, 62, 63].

Keeping the patient safe during care transitions seemed to be a shared understanding between health care providers across the STCF and HCS and influenced IPC. The safety issue involved the patient’s physical as well as psycho-social needs and condition at home.

Paradoxically, keeping the patient safe did not seem to be an issue regarding patient in transition to LTCF to others than the health care providers in the LTCF. According to White paper 38 [64], the municipal prioritisation principles be the same as the specialised health and care services principles: usefulness, seriousness, and resources. Furthermore, in line with the Blankholm Committee [65] coping in relation to the principles usefulness and seriousness is added. These principles may function well regarding patient with the potential of managing at home after a stay, or recurring, regular stays at the STCF. Still, our findings suggest that these principles do not consider patients in transition to LTCF. To sufficiently assist and support older frail patients, Heggestad and colleagues [62, 66] propose to include maintenance of older persons basic needs and safety as a minimum standard to fulfil for all patients.

The nursing staff and the physiotherapists in particular, regarded IPC between the STCF and the HCS necessary to improve the quality of their assistance. To assist a patient moving home comprised supporting the patient towards coping and coming to terms with his/her new situation [24]. Supper et al. [39], also found, in their review study of IPC in primary health care, that the individual health care provider perceived IPC an “opportunity to improve quality of care and to develop new professional fields” (p. 716). That the two auxiliaries in the HCS compensated for the vulnerable physiotherapy services, may illustrate that the auxiliaries developed new professional fields. It contributed to immediate and close follow-up of patients at key points in their transition. I.e., shortly after the patient arrived home, the auxiliary assisted the patient in physical exercises prescribed by the physiotherapist. Although this contributed to efficient and necessary assistance, we question how favourable such IPC is. The findings indicate that the municipality lacked resources and that the physiotherapists supervised the auxiliaries to compensate for their own limitations. However, physiotherapists have special competence that auxiliaries do not have. It is unclear from the findings whether this blending of competence was based on thorough professional judgement or mainly on limited municipal resources. It is paramount that health care providers across levels and professions carefully consider which competence to blend and at what costs. Due to limited professional resources, the GP at the STCF sent stroke patients for rehabilitation outside the municipality. This ensured competent assistance of these patients, yet higher costs for the municipality.

At the LTCF, some registered nurses expressed concerns about feeling their work less appreciated in the municipality. The lack of regular and formalised interaction with colleagues across facilities may emphasise the notion of caring for older patients in LTCF as both “a domain outside the main sphere of nursing” ([67], p. 332), and “a domain outside the sphere of IPC “. This sense of devalue among registered nurses may ultimately contribute to difficulties in recruitment of qualified nursing staff in LTCFs. As demonstrated by the findings, neither the GP at the LTCF nor the registered nurses talked about collaborating with each other during patients’ transition to LTCF. If the GP showed little interest in discussing and sharing with registered nurses, one may wonder if this added to the registered nurses’ sense of devalue. Thus, one may speculate that these omissions of each other by the two professional groups demonstrate power inequalities taken for granted and unspoken of. Paraphrasing Foucault “forms of oppression and domination that remain invisible run the risk of becoming the norm” ([68], p. 2).

During transition to LTCF, health care providers across professions and facilities need collaborate to provide comprehensive health and care services for the patients and their next of kin in this period of upheaval [5, 6, 24, 25]. According to White paper 15 ([9], p. 27), municipalities need to facilitate smoother and planned care transitions from home to nursing home. National guidelines [1, 9] and studies [69–71], stress that municipal health care providers need to expand and develop knowledge and competence to detect early signs of patients at risk.

In the HCS, the use of whiteboards facilitated IPC and promoted shared understandings and blending of competence among health care providers during patients’ transitions. However, unlicensed assistants challenged IPC. The licensed nursing staff compensated for the assistants, which may interfere with professional standards, ethics, and prioritizations. Norheim and Thoresen [72] also found that registered nurses experienced difficulties in using their competence at the right place and right time due to inappropriate division of labour and lack of resources. That the licensed nursing staff documented in writing what assistants who did not have a password reported to them, may contribute to important information about the patients is omitted. Assistants have no formal training in health care and may not notice and pass on vital information about the patients. Furthermore, they may forget details when sharing their observations orally [6]. Also, assistants are at the bottom of the staff hierarchy, and may take for granted that what they know or notice, everybody else knows, too, so there is no need to pass on this information [6]. Finally, it may be challenging for the licensed nurse to write what is told to her because it is time-consuming, and the information may be imprecise. However, these meeting points between the licensed and unlicensed nursing staff may be an opportunity to support and supervise the assistants. Complex adaptive system theory stresses that every agent in the organisation matters [6, 73]. As long as municipalities depend on unlicenced assistants, measures need be taken to secure that their contributions are valued and used for the benefit of the patients.

Studies highlight that GPs are central in IPC during care transitions [2, 38, 74, 75]. Yet, as indicated by the findings, neither most GPs nor their colleagues talked about this centrality. At the medical centre, the GPs’ involvement in IPC during patients’ transitions varied and seemed to depend on personal, professional values as well as organisational and geographical circumstances. Steihaug et al. [3], also found that GPs’ collaborative patterns depended on the GP’s individual priorities, consideration of importance, and practical barriers related to organisation. Similarly, studies show variation in the involvement of GPs during care transitions. For instance, Skrove et al. [76], found that GPs did not want to be involved in the implementation of care pathways, while Vassbotn et al. [38], found that GPs experienced being left out from important decision-making. Both perspectives appeared relevant in our findings.

Geographical locations challenged collaboration between the nurses in the HCS and the GPs at the medical centre. Studies suggest that spatial proximity and co-locations can improve the quality and quantity of communication between actors [77, 78] Studies [51, 79] and White Paper 7 [2] suggest meeting points between the specialist and the municipal health and care services. The findings in the current study suggest interprofessional meeting points involving the LTCF where the physician at the facility participates with the nursing staff and physiotherapists. At these meetings, health care providers acquire knowledge about each other’s work contexts, and may develop relationships and mutual understanding of each other’s roles and responsibilities. Health care providers and their leaders should also discuss which competencies to blend across professions (assistants included), and which not to blend, to ensure professionally sound and equitable health and care services. Moreover, power mechanisms at structural, professional, and personal levels should be articulated to improve collaboration between professional groups during this time of upheaval [25] for the patients.

### Limitations

The study is limited to one rural municipal context and is not altogether transferable to other municipal settings. Yet, since no studies so far has investigated this phenomenon, this study may give direction to future studies.

### Further research

Further studies need to incorporate patients’ and next of kin’s experiences during older patients transitions in the municipality. Studies with larger samples and different designs in urban as well as rural contexts could elucidate more knowledge on this topic.

## Conclusion

Prioritising and facilitating inter-professional collaboration between the short-term care facility and the home care services, contributed to health care providers experiencing doing a proper and safe patient assistance. Yet, this priority was at a cost: health care providers in the long-term care facility, and in particular registered nurses felt squeezed and of less worth in the municipal health and care services. It was a strain on them to experiencing unplanned and often rushed patient transition into long-term care facility. To focus on municipal inter-professional and inter-facility collaboration during patients in transition to long-term care placement is vital to maintain the patients, and the health care providers working in these facilities.

## Supplementary Information

**Additional file 1.**

**Additional file 2.**

## Data Availability

Approval from NSD requires that the transcriptions of interviews are kept in locked files, accessible only by the authors. The interview guide is supplied as Additional file 1. First author (ME) may be contacted if access to transcribed data is required.

## Abbreviations

IPC: Interprofessional collaboration
STCF: Short-term care facility
HCS: Home care services
LTCF: Long-term care facility
FG: Focus group interviews
