# Supplementary material for: Municipal healthcare professionals’ interprofessional collaboration during older patients’ transitions in the municipal health and care services: a qualitative study
Source: BMC Health Serv Res. 2022 Jul 15;22:918. doi: 10.1186/s12913-022-08226-5 (PMC9284810; doi:10.1186/s12913-022-08226-5)
Supplement: Supplementary file 1 — Additional file 1. [file 12913_2022_8226_MOESM1_ESM.docx]

**Additional file 1**

Municipal healthcare professionals interprofessional collaboration during older patients transitions in the municipal health and care services: a qualitative study

**Semi-structured interview guide**

- Your experiences with IPC (intra- facility and across facilities) during older patients’ transition within the MHCS.
- Describe what contributes to good IPC during older patients in transition
- Describe what contributes to insufficient or lack of collaboration/IPC and possible barriers (at individual as well as organizational levels).
- Describe environments (circumstances and situations) that encourage IPC (and the opposite).
- Local culture: does knowing the patient matter and influence IPC?
- Describe characteristics of a good team worker during older patients’ transition (and the opposite) and how it influences IPC
- Describe characteristics/facilitators of team environments that encourage IPC (structural/organizational contexts and circumstances)
- Describe patients and next of kin’s influence on IPC
