# Supplementary material for: Municipal healthcare professionals’ interprofessional collaboration during older patients’ transitions in the municipal health and care services: a qualitative study
Source: BMC Health Serv Res. 2022 Jul 15;22:918. doi: 10.1186/s12913-022-08226-5 (PMC9284810; doi:10.1186/s12913-022-08226-5)
Supplement: Supplementary file 2 — Additional file 2. [file 12913_2022_8226_MOESM2_ESM.docx]

**Additional file 2**

**Additional file 2: Themes and categories:** Municipal healthcare professionals interprofessional collaboration during older patients transitions in the municipal health and care services: a qualitative study

Sub-themes and themes regarding what influenced municipal IPC during older patients’ transition in the municipality

Abbreviations: Health care provider: HCP

| Sub-theme | Themes |  |
| --- | --- | --- |
| Common patient diseases, needs and treatment |  |  |
| Assisting patients with their psychosocial needs a professional standard |  |  |
| Prioritizing rehab patients and possibility of staying home longer | **Theme 1** |  |
| Patients insisting on moving home | Patient situations that influenced IPC during older patients’ transitions in the municipality related to prioritisations, expectations, relationships, dilemmas |  |
| Next of kin expecting too much assistance – alliances between HCPs |  |  |
| next of kin deciding on patient’s behalf – promoted close IPC GP and RNs |  |  |
| Planned transitions stimulated IPC between STCF and HCS, **good circle**: IPC contributed to planned transitions |  |  |
| (Due to patient- and next of kin situation and circumstances): Rushed and unplanned transitions challenged IPC  **Patients in transition to LTCF not prioritised** no formalised IPC across facilities. Some professionals not interested (GP and physios) |  |  |
| Patients unable to stay home despite comprehensive IPC support contributed to planned and smooth transition to LTCF |  |  |
|  |  |  |
|  |  |  |
|  | **Theme 2** |  |
| HCPs’ professional and personal attitudes positive to IPC on behalf of patients – paramount that patients are safe  Regarded IPC essential in transition |  |  |
| IPC depend on person and group dynamics: good “chemistry” between HCPs vital |  |  |
| Municipal prioritisations and limited municipal resources strong impact on IPC |  |  |
| Admission team meeting and its members decisive in making prioritizations and encouraging IPC within facilities and across especially the HCS and the STCF  - decisions made at the meetings influenced IPC within and across facilities |  |  |
| Admission team members close to their front-line staff encouraged collaboration both ways |  |  |
| Different facilities with different functions, professional resources and contexts influenced IPC. (Professional roles and functions influenced IPC; Clear roles and expectations within and between facilities facilitated IPC)    -**HCS** – assistants challenged IPC, difficult to get in touch with patients’ GP  -**LTCF** – poor relationship with GP, compensated with strong intra-facility collaboration between nursing staff  -**STCF-** clear roles, expectations, and relationship between health care providers and good relationship with GP at the facility. Shared interests and professional standards, aim: “culture of generosity”  - **GP** not at the STCF when IPC meetings were held  - Use of unlicensed assistants challenged and stimulated IPC – differed between facilities |  |  |
| Formalized meeting points across STCF and HCS |  |  |
| No formalized meeting points between the LTCF and the other facilities – IPC with LTCF downgraded |  |  |
| Random IPC between LTCF and other municipal health care facilities influenced negatively on registered nurses’ self-esteem and sense of worth | Professional, Personal and Practical Circumstances that influenced IPC during older patients’ transition in the municipality related to competence, roles, expectations, compensations, prioritisations, and local contexts |  |
| GP and nursing staff at the LTCF did not talk about collaborating with each other |  |  |
| Nursing staff in LTCF compensated lack of IPC within and across facilities with close intra-facility nursing staff collaboration |  |  |
| Computer program, whiteboards, and telephone significant in IPC – not everyone read documentation on computer |  |  |
| In HCS, two auxiliaries compensated and complemented vulnerable physiotherapy service |  |  |
| GPs expectations and involvement regarding IPC varied; professional, structural, and geographical reasons |  |  |
| The taken for granted challenged IPC  -Communication between HCP: forget to inform each other – HCPs used to patients in care transition – forget that moving to LTCF is once in a lifetime for the patients  -also a danger that HCPs take for granted circumstances |  |  |
